# Antibodies to Domain I β_2_-Glycoprotein 1 in Patients with Antiphospholipid Syndrome and Systemic Lupus Erythematosus

**DOI:** 10.1134/S1607672923700278

**Published:** 2023-10-13

**Authors:** T. M. Reshetnyak, F. A. Cheldieva, M. V. Cherkasova, S. I. Glukhova, A. M. Lila, E. L. Nasonov

**Affiliations:** 1 Nasonova Research Institute of Rheumatology, Moscow, Russia; 2 Russian Medical Academy of Continuous Professional Education of the Ministry of Healthcare of the Russian Federation, Moscow, Russia; 3 Sechenov First Moscow State Medical University of the Ministry of Health Care of Russian Federation (Sechenov University), Moscow, Russia

**Keywords:** antiphospholipid antibodies, antiphospholipid syndrome, antibodies to domain I β_2_ glycoprotein 1, pregnancy morbidity, thrombosis

## Abstract

The role of antiphospholipid antibodies (aPL), which are not included in the Sydney diagnostic criteria, in antiphospholipid syndrome (APS) and systemic lupus erythematosus (SLE) is poorly understood. The aim of this study was to determine the clinical significance of IgG antibodies for domain 1 of β_2_-glycoprotein 1 (β_2_-GP1), IgG anti-β_2_-GP1DI, in patients with APS with and without SLE. The study included 187 patients with APS with or without SLE, 49 patients formed the comparison group, and 100 apparently healthy individuals formed the control group. IgG/IgM antibodies to cardiolipin (aCL) and IgG/IgM anti-β_2_-GP1 were determined by enzyme immunoassay (ELISA) in patients with or without APS, and IgG anti-β_2_-GP1DI was determined by chemiluminescence assay (CLA) in all patients and controls. IgG anti-β_2_-GP1DI was detected in 37 (71%) of 52 patients with primary APS (PAPS), in 6 (50%) of 12 patients with probable APS, in 42 (71%) of 59 patients with SLE + APS, in 17 (26%) of 64 patients with SLE, in 1 (2%) of the comparison group, and in none of the control group. IgG anti-β_2_-GP1DI was significantly associated with PAPS and SLE + APS compared with the patients with SLE (*p* = 0.0002 and 0.0001, respectively). The association of IgG anti-β_2_-GP1DI with clinical manifestations of APS (thrombosis (*p* = 0.001) and obstetric pathology (*p* = 0.04)) was detected. There was a significant association of IgG anti-β_2_-GP1DI with arterial thrombosis (*p* = 0.002) and with late gestational obstetric pathology (*p* = 0.01). High specificity of IgG anti-β_2_-GP1DI depending on the diagnosis and clinical manifestations of APS despite low sensitivity was noted: specificity was 84% for thrombosis, 94% for obstetric pathology, and 89% for APS. Isolated IgG anti-β_2_-GP1DI positivity was reported in 2% of 50 aPL-negative patients and was not associated with APS manifestations. The frequency of IgG anti-β_2_-GP1DI detection was higher in the patients with APS compared to the patients with SLE, comparison group, and control (*p* < 0.05). Positive IgG anti-β_2_-GP1DI values were significantly associated with thrombotic complications and with obstetric pathology (*p* = 0.002 and *p* = 0.01, respectively). Specificity of IgG anti-β_2_-GP1DI for APS and its clinical manifestations (thrombosis and obstetric pathology) was higher than sensitivity (89, 94, and 84%, respectively).

## INTRODUCTION

Antiphospholipid syndrome (APS) is a symptom complex represented by recurrent thromboses (arterial and/or venous) and obstetric pathology (more often, fetal loss syndrome) associated with the synthesis of antibodies that react with a wide range of phospholipids (PLs) and PL-binding proteins [1–4]. For the diagnosis of APS, classification criteria were developed that include, along with clinical manifestations (thromboses and obstetric pathology), the detection of antibodies to PLs (aPLs), including IgG/IgM antibodies to cardiolipin (aCL), IgG/IgM antibodies to β_2_-glycoprotein 1 (anti-β_2_-GP1), and lupus anticoagulant (LA) [5, 6]. The determination of the listed aPLs is important not only for confirming the diagnosis of APS, but also for assessing the risk of recurrent thromboses [7–9]. To improve the laboratory diagnosis of APS and to stratify the risk of developing thromboses, the so-called “non-criteria” aPLs, which include antibodies primarily to domain I of β_2_-glycoprotein 1 (anti-β_2_-GP1DI) [10–25]. β_2_-Glycoprotein 1 (β_2_-GP1), or apolipoprotein H, which is a “cofactor” in the determination of aCL, consists of five domains [23–25]. Antibodies recognizing a specific epitope (G40–R43) of domain I are associated with the APS development [11–13, 15, 16, 19, 22].

The aim of this research was to study the clinical significance of IgG antibodies to domain I of β_2_-glycoprotein 1 in antiphospholipid syndrome in combination with systemic lupus erythematosus and in primary antiphospholipid syndrome.

## MATERIALS AND METHODS

The main group included 187 patients with primary APS (pAPS), APS in combination with systemic lupus  erythematosus (SLE), or SLE without APS: 52 patients with pAPS, 12 with “probable” APS, 59 with SLE + APS, and 64 with SLE without APS (Table 1). The control group consisted of 100 healthy donors. The comparison group included 49 outpatients with a referral diagnosis of APS, which was not confirmed: seven of them had thrombosis without aPL, ten had rheumatoid arthritis, fifteen Behçet's disease, twelve had systemic sclerosis, two had pregnancy, two had polymyositis, and one had Burger’s endarteritis. The diagnosis of SLE complied with the criteria of the American College of Rheumatology 1997 [26], and the diagnosis of APS complied with the international criteria [5]. In the absence of signs of other diseases, patients meeting the criteria for APS were diagnosed with pAPS. In case of persistent aFL positivity and/or in the presence of non-criteria manifestations of the disease (livedo, thrombocytopenia, cerebral microangiopathy, etc.) with exclusion of other rheumatic diseases and diseases contributing to aFL production, probable APS was diagnosed.

**Table 1.** Characteristics of patients included in the study

Parameters		PAPS, *n* = 52	Probable APS, *n* = 12	SLE + APS, *n* = 59	SLE, *n* = 64
Average age, Me[25; 75 percentiles], years		38.0 [32.5; 43.0]	34.0 [29.5; 44.5]	40.0 [33.0; 47.0]	31.5 [24.0; 40.5]*
Duration of disease, Me[25; 75 percentiles], years		8.4 [3.1; 13.5]	0.9 [0.3; 2.1]**	15.0 [7.3; 21.0]	4.0 [1.6; 8.0]***
Sex: male/female, abs		29 (56)/23 (44)	10 (83)/2 (17)	47 (80)/12 (20)	56 (87.5)/8 (12.5)
History of thrombosis****, abs (%)	arterial	26 (50)	1 (8)	27 (46)	4 (6)
	venous	31 (60)	1 (8)	36 (61)	12 (19)
Obstetric pathology *, *n* (%)/*n*		19(95)/20	1 (50)/2	26 (84)/31	7 (44)/16
IgG aCL, *n* (%)		37 (71)	7 (58)	39 (66)	8 (12.5)
IgM aCL, *n* (%)		12 (23)	3 (25)	10 (17)	7 (11)
IgG anti-β_2_-GP1, *n* (%)		36 (69)	7 (58)	44 (75)	9 (14)
IgM anti-β_2_-GP1, *n* (%)		12 (23)	5 (42)	12 (20)	7 (11)
Lupus anticoagulant*****, *n* (%)/*n*		7 (87.5)/8	8 (89)/9	10 (71)/14	5 (21)/24

* *p* = 0.01 compared to systemic lupus erythematosus with antiphospholipid syndrome; ** *p* = 0.01 compared to primary antiphospholipid syndrome and *p* = 0.000001 compared to systemic lupus erythematosus with antiphospholipid syndrome; *** *р* = 0.000001 compared to systemic lupus erythematosus with antiphospholipid syndrome; **** obstetric pathology was calculated in women who had pregnancy in their disease course; the numerator is the number and percent of women with obstetric pathology, and the denominator is the number of women who had pregnancy in their disease course; ***** lupus anticoagulant study was performed in patients who did not receive anticoagulant therapy; the numerator is the number and percent of patients with positive lupus anticoagulant, and the denominator is the number of patients who had lupus anticoagulant determination; IgG/IgM aCL and IgG/IgM anti-β_2_-GP1 were determined by enzyme immunoassay.

All patients were assayed for IgG/IgM aCL and IgG/IgM anti-β_2_-GP1 by enzyme immunoassay (ELISA) on an Alegria automatic analyzer (Orgentec Diagnostika GmbH, Germany) with a reagent kit for antibody detection (Orgentec Diagnostika GmbH, Germany) according to the manufacturer’s instructions.

IgG anti-β_2_-GP1DI was determined by chemiluminescence analysis (CLA) using the BIO-Flash® instrument (Biokit S.A., Spain). The QUANTA Flash® kit (United States) was used to determine anti-β_2_-GP1DI IgG. IgG anti-β_2_-GP1DI were measured in chemiluminescent units (CU). Levels >19 CU were considered positive, as recommended by the reagent manufacturer.

The study of LA was performed on an automatic coagulometer manufactured by Siemens Healthcare (Germany) using screening (LA1) and confirmatory (LA2) tests. LA was determined only in 55 patients (in the remaining 132, LA could not be determined due to anticoagulant therapy). When statistically processing the results, we used the following indices to describe quantitative variables: median (Me), 25th and 75th percentiles, and frequency for qualitative variables. Differences were considered statistically significant at *p* ≤ 0.05. For quantitative variables, a normal distribution test was performed. For the parameters whose distribution differed from normal when comparing two groups, the Mann–Whitney test was used.

The obtained results were statistically processed using the χ^2^ (Pearson’s test). ROC curves were built using the IBM SPSS Statistics 13.0 for Windows software package (IBM Corporation, United States). The positive predictive value (PPV) was calculated using the formula [27]:

$$\frac{{{\text{Number of true positives}}}}{{{\text{Number of true positives}} + {\text{number of false positives}}}}$$

The negative predictive value (NPV) was calculated using the formula [27]:

$$\frac{{{\text{Number of true negatives}}}}{{{\text{Number of true negatives}} + {\text{number of false negatives}}}}$$

The odds ratio (OR) for a positive test result was calculated using the formula [28]:

$$\frac{{{\text{Sensitivity}}}}{{1 - \,\,{\text{Specificity}}}}$$

OR equal to 1 means that the probability of a positive test result in a patient is the same as the probability of a positive test result in a healthy person.

The statistical data analysis package Statistica 10.0 for Windows (StatSoft Inc., United States), SPSS Statistics 13.0 for Windows (IBM Corp., United States), and VassarStats (United States) software was used.

## RESULTS AND DISCUSSION

**Fig. 1.**
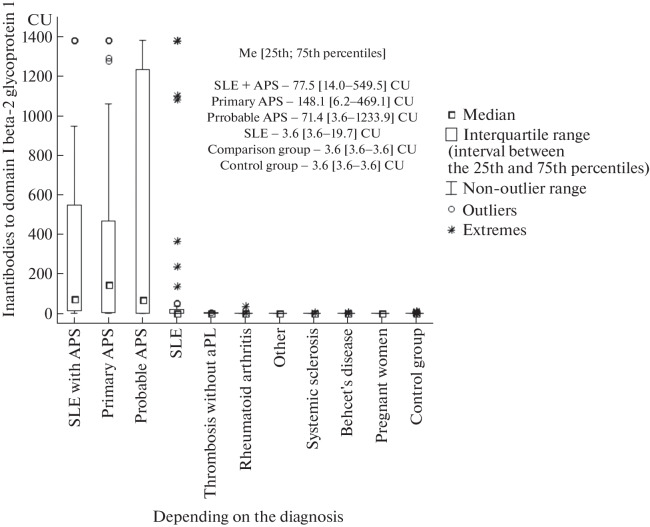
Levels of IgG anti-β_2_-GP1DI. Designations: SLE, systemic lupus erythematosus; APS, antiphospholipid syndrome; aPL, antiphospholipid antibodies; other, two patients with polymyositis (one of whom had thrombosis) and one patient with Buerger’s endarteritis.

IgG anti-β_2_-GP1DI were detected in 37 (71%) of 52 patients with pAPS; in 6 (50%) out of 12 with probable APS, in 42 (71%) of 59 with SLE + APS, in 17 (26%) of 64 with SLE, in 1 (2%) from the comparison group (a patient with a high activity of rheumatoid arthritis), and in none of the subjects in the control group. In the patients with pAPS and SLE + APS, positive values of anti-β_2_-GP1DI IgG were detected statistically significantly more often than in the patients with SLE without APS (*p* = 0.0002 and *p* = 0.0001, respectively). The levels of IgG anti-β_2_-GP1DI in patients with pAPS, probable APS, SLE + APS, and SLE were statistically significantly higher than in the control group (*p* < 0.000001, *p* = 0.03, *p* < 0.000001, and *p* = 0.02, respectively) (Fig. 1). In the patients with pAPS and SLE + APS, the levels of IgG anti-β_2_-GP1DI were statistically significantly higher than in the patients with SLE without APS (*p* = 0.001 and *p* = 0.000005, respectively) and in the comparison group (*p* < 0.05).

Depending on the detection of IgG anti-β_2_-GP1DI, patients with APS were divided into two groups: the first group included patients with positive results of anti-β_2_-GP1DI IgG determination; the second group, with negative results (Table 2).

**Table 2.** Clinical signs of antiphospholipid syndrome depending on the results of the study of IgG antibodies to domain I of beta-2 glycoprotein 1 (IgG anti-β_2_-GP1DI)

Parameter		IgG anti-β_2_-GP1DI ( + ), *n* (%) (*n* = 102)	IgG anti-β_2_-GP1DI (–), *n* (%) (*n* = 85)	χ^2^; *p* OR [95% CI]
Thrombosis (total)	yes	72 (71)	41 (48)	9.69; 0.001 2.63 [1.42–4.76]
	no	30 (29)	44 (52)	
Arterial thrombosis	yes	41 (40)	17 (20)	8.84; 0.002 2.70 [1.38–5.26]
	no	61 (60)	68 (80)	
Venous thrombosis	yes	49 (48)	31 (36)	2.53; 0.11 1.61 [0.90–2.94]
	no	53 (52)	54 (64)	
Pregnancy morbidity*	yes	32(86); *n* = 37	21 (66); *n* = 32	4.19; 0.04 3.44 [1.02–11.11]
	no	5 (14)	11 (34)	

Total, total number of thrombosis regardless of localization; *n*, number of patients; χ^2^, agreement criterion; *p*, significance; OR, odds ratio; CI, confidence interval; IgG, immunoglobulin G; * pregnancy morbidity calculated from the number of women who had pregnancy against the background of the disease.

The detection of IgG anti-β_2_-GP1DI was associated with thrombosis and obstetric pathology; the risk of developing clinical manifestations of APS was 2.63 and 3.44, respectively (Table 2). There is a statistically significant association between the detection of anti-β_2_-GP1DI IgG and the development of arterial thrombosis, including acute cerebrovascular accident (*p* = 0.003), as well as with the development of obstetric pathology (*p* = 0.04), primarily eclampsia/preeclampsia and placental insufficiency in late gestation (*p* = 0.01).

In general, the detection of IgG anti-β_2_-GP1DI was statistically significantly associated with APS (*p* < 0.0001). The risk of developing APS in the patients with positive IgG anti-β_2_-GP1DI values was 5.88 times higher than in the patients with negative results of determination of these antibodies.

**Fig. 2.**
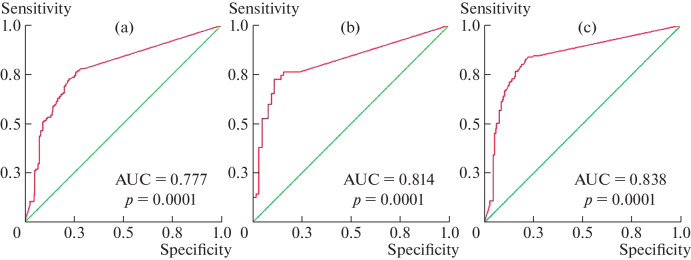
ROC curves for IgG anti-β_2_-GP1DI depending on thrombosis (a), obstetric pathology (b), and reliable antiphospholipid syndrome (c). Designations: AUC, area under curve; *p*, significance.

The sensitivity and specificity of IgG anti-β_2_-GP1DI was assessed from ROC curves (Fig. 2) depending on the presence of thrombosis, obstetric pathology, and reliable APS. The area under the ROC curve depending on thrombosis was 0.777 (95% CI, 0.720–0.835); on obstetric pathology, 0.814 (95% CI, 0.732–0.897); and on the diagnosis of APS, 0.838 (95% CI, 0.787–0.889 ) (*p* = 0.0001).

Sensitivity and specificity, positive and negative predictive values (PPV and NPV), and odds ratio (OR) for APS and its clinical manifestations (thrombosis and obstetric pathology) were calculated from the ROC curves (Table 3).

**Table 3.** Results of ROC analysis

Parameter		Thrombosis	Pregnancy morbidity	APS diagnosis
IgG anti-β_2_-GP1DI	Sensitivity (%)	54	53	71
	Specificity (%)	84	94	89
	Likelihood ratio test	3.705	12.500	5.461
	PPV	70	86	76
	NPV	77	72	86

APS, antiphospholipid syndrome; IgG, immunoglobulin G; IgG, anti-β_2_-GP1DI, IgG antibodies to domain I of beta-2 glycoprotein 1; PPV, positive predictive value; NPV, negative predictive value.

The detection of IgG anti-β_2_-GP1DI for obstetric pathology had a higher specificity than for thrombosis and a reliable diagnosis of APS. The probability of obstetric pathology in the patients positive for IgG anti-β_2_-GP1DI was 12.5 times higher in terms of OR and by 86% in terms of PPV than in their absence (Table 3). The risk of thrombosis in the IgG anti-β_2_-GP1DI-positive patients was 3.7 times higher than in the absence of these antibodies. The probability of APS in the presence of IgG anti-β_2_-GP1DI was 5.461 times higher than in their absence. The probability of having APS at IgG anti-β_2_-GP1DI positivity was 71%, and the probability of the absence of APS at negative results of IgG anti-β_2_-GP1DI determination was 87%.

In 50 (29%) of 187 patients of the main group, aPL included in the APS criteria were absent. Isolated IgG anti-β_2_-GP1DI positivity in the patients without classical aPL occurred in 2% (in 1 patient out of 50) in CLA. In one SLE patient with negative results of classic aPL detection in CLA, the level of IgG anti-β_2_-GP1DI was 36.0 CU. She had no clinical manifestations of APS and pancytopenia was detected as part of SLE activity.

Anti-β_2_-GP1 is a heterogeneous population of autoantibodies that react with β_2_-GP1 itself or with the β_2_-HP1 complex with anionic PLs [23, 29]. β_2_‑GP1 is a highly conserved plasma protein (326 amino acids) comprising five domains (short consensus repeat) and, depending on the nature of binding to anionic PLs, forms circular or linear forms [25, 29, 30]. Domain 5 contains several hydrophobic residues and is highly flexible, due to which it is able to interact with anionic PLs on the plasma membrane. This leads to the release of a cryptic epitope in domain I, which has a potential “autoantigenic” activity [30, 31], because autoantibodies present in the sera of APS patients react more strongly with this epitope than with antigenic determinants present in other β_2_-GP1 domains [32–36]. According to experimental studies, the region of glutamine 40-Arginine 43 (G40–R43) in domain I of β_2_-GP1 is critical in terms of inducing the synthesis of antibodies that cause thrombosis and obstetric pathology [37]. It is assumed that, since β_2_-GP1DI has a high homology with the extracellular region of the Toll receptor (TLR4), the pathogenic potential of anti-β_2_-GP1DI may be due to their ability to activate the signaling pathway including TLR4 and NF-κB (nuclear factor kappa B) [38].

Currently, ELISA and CLA are used to detect anti-β_2_-GP1DI [39, 40]. According to our data, in CLA, IgG anti-β_2_-GP1DI was detected in 71% of patients with primary APS, in 50% of patients with probable APS, and less often (26%) in patients with SLE. In studies by other authors, IgG anti-β_2_-GP1DI was found in 31–48.6% of patients with primary APS and in 46% of patients with probable APS [39, 41]. According to meta-analysis data, the detection rate of anti-β_2_-GP1DI IgG in patients with APS was 44.0% [42]. In another study, anti-β_2_-GP1DIs were detected in 69% of patients with primary APS and in 56% of patients with probable APS. According to a systematic review, a statistically significant association between the detection of IgG anti-β_2_-GP1DI and the development of thrombosis was found in four out of five studies [16]. The results of a prospective study suggest that, in patients with APS, an increase in the concentration of IgG anti-β_2_-GP1DI is an independent risk factor for thrombosis [43]. Data on the relationship between the detection of IgG anti-β_2_-GP1DI and the development of obstetric pathology are contradictory. According to Zhang et al. [41], who observed 229 patients (35 with primary APS, 51 with secondary APS, 30 with thrombosis not associated with APS, 32 with pathology of pregnancy not associated with APS, 42 with SLE, and 39 in the control group), no association between the detection of IgG anti-β_2_-GP1DI and obstetric pathology was found. At the same time, according to the results of our study, a history of obstetric pathology was revealed more often in the women with IgG anti-β_2_-GP1DI than in the women without IgG anti-β_2_-GP1DI (*p* = 0.04). The association between the detection of IgG anti-β_2_-GP1DI and obstetric pathology in late gestation was confirmed by other authors [44]. In addition, in a retrospective analysis of 135 women with obstetric pathology with persistently positive moderate or high levels of IgG anti-β_2_-GP1DI, without concomitant systemic autoimmune diseases and with at least one previous pregnancy, the detection of IgG anti-β_2_-GP1DI was a predictor of preeclampsia (OR = 2.4, 95% CI 1.2–5.0; *p* = 0.017) [18]. The association between the detection of IgG anti-β_2_-GP1DI and the development of obstetric pathology in late gestation, which we found, indicates the advisability of a dynamic study of these antibodies in the second half of gestation, for the timely prevention of complications.

The question of the sensitivity and specificity of IgG anti-β_2_-GP1DI for the diagnosis of APS remains open. High titers of IgG anti-β_2_-GP1DI are often found in patients with “triple positivity” for aPL [11–15] and are associated with thrombosis and obstetric pathologies [16–18]. However, according to some authors, the detection of IgG anti-β_2_-GP1DI is not an independent risk factor for APS compared to aPLs, which are included in the criteria for APS [11, 12, 14, 19]. At the same time, according to Liu et al. [45], IgG anti-β_2_-GP1DI is a promising biomarker with a high specificity (97%) and a moderate sensitivity (64%) for diagnosing APS. The sensitivity and specificity of IgG anti-β_2_-GP1DI for the diagnosis of APS, according to our data, were 71 and 89%, respectively. In assessing the clinical manifestations of APS, the specificity was higher than the sensitivity for thrombosis and amounted to 54%, specificity was 84%; for obstetric pathology, l53 and 94%, respectively. It is also assumed that the determination of IgG anti-β_2_-GP1DI may be a useful “second-line” test in patients with an isolated increase in the concentration of anti-β_2_-GP1DI or “double positivity” for aPL to identify the “pathogenic” population of anti-β_2_-GP1DI [9, 46], since an isolated increase in the concentration of these antibodies is not associated with the development of thrombosis [22].

Taken together, the results of our study suggest the potential value of IgG anti-β_2_-GP1DI detection in revealing APS patients with a high risk of thrombosis and obstetric pathology.

## References

[1] Ruiz-Irastorza G., Crowther M., Branch W., Khamashta M.A. (2010). Antiphospholipid syndrome. Lancet.

[2] Nasonov E.L. (2004). *Antifosfolipidnyi sindrom* (Antiphospholipid Syndrome).

[3] Reshetnyak, T.M., Cheldieva, F.A., Nurbayeva, K.S., Lila, A.M., and Nasonov, E.L., Antiphospholipid syndrome: diagnosis, development, and therapy, *Tromboz, Gemostaz Reologia*, 2020, no. 4, pp. 4–21.

[4] Reshetnyak T.M. (2014). Antiphospholipid syndrome: diagnosis and clinical manifestations (a lecture), *Nauchno-Prakt.*. Revmatol..

[5] Miyakis S., Lockshin M.D., Atsumi T. (2006). International consensus statement on an update of the classification criteria for definite antiphospholipid syndrome (APS). J. Thromb. Haemost.

[6] Devreese K.M.J., Ortel T.L., Pengo V., de Laat B., Subcommittee on lupus anticoagulant/antiphospholipid antibodies (2018). Laboratory criteria for antiphospholipid syndrome: communication from the SSC of the ISTH. J. Thromb. Haemost..

[7] Pengo V., Ruffatti A., Legnani C. (2010). Clinical course of high-risk patients diagnosed with antiphospholipid syndrome. J. Thromb. Haemost..

[8] Vandevelde A., Devreese K.M.J. (2022). Laboratory diagnosis of antiphospholipid syndrome: insights and hindrances. J. Clin. Med..

[9] Devreese K.M.J., Zuily S., Meroni P.L. (2021). Role of antiphospholipid antibodies in the diagnosis of antiphospholipid syndrome. J. Transl. Autoimmun.

[10] de Laat B., Derksen R.H., Urbanus R.T., de Groot P.G. (2005). IgG antibodies that recognize epitope Gly40–Arg43 in domain I of beta 2-glycoprotein I cause LAC, and their presence correlates strongly with thrombosis. Blood.

[11] Yin D., de Laat B., Devreese K.M.J., Kelchtermans H. (2018). The clinical value of assays detecting antibodies against domain I of β_2_-glycoprotein I in the antiphospholipid syndrome. Autoimmun. Rev..

[12] De Craemer A.S., Musial J., Devreese K.M. (2016). Role of anti-domain 1-β_2_ glycoprotein I antibodies in the diagnosis and risk stratification of antiphospholipid syndrome. J. Thromb. Haemost..

[13] Pengo V., Ruffatti A., Tonello M. (2015). Antiphospholipid syndrome: antibodies to domain 1 of beta2-glycoprotein 1 correctly classify patients at risk. J. Thromb. Haemost.

[14] Yin D., Chayoua W., Kelchtermans H. (2020). Detection of anti-domain I antibodies by chemiluminescence enables the identification of high-risk antiphospholipid syndrome patients: a multicenter multiplatform study. J. Thromb. Haemost.

[15] Nascimento I.S., Radin M., Gândara A.P.R., Sciascia S., de Andrade D.C.O. (2020). Global antiphospholipid syndrome score and anti-ß2-glycoprotein I domain I for thrombotic risk stratification in antiphospholipid syndrome: a four-year prospective study. Lupus.

[16] Radin M., Cecchi I., Roccatello D. (2018). Prevalence and thrombotic risk assessment of Anti-β_2_ glycoprotein I domain I antibodies: a systematic review. Semin. Thromb. Hemost.

[17] Zuily S., de Laat B., Guillemin F. (2020). Anti-domain I β_2_-glycoprotein I antibodies and activated protein C resistance predict thrombosis in antiphospholipid syndrome: TAC(I)T study. J. Appl. Lab. Med.

[18] Chighizola C.B., Pregnolato F., Andreoli L. (2018). Beyond thrombosis: anti-β_2_GPI domain 1 antibodies identify late pregnancy morbidity in anti-phospholipid syndrome. J. Autoimmun..

[19] Iwaniec T., Kaczor M.P., Celińska-Löwenhoff M. (2017). Clinical significance of anti-domain 1 β_2_-glycoprotein I antibodies in antiphospholipid syndrome. Thromb. Res.

[20] Pignatelli P., Ettorre E., Menichelli D. (2020). Seronegative antiphospholipid syndrome: refining the value of “non-criteria” antibodies for diagnosis and clinical management. Haematologica.

[21] Bradacova P., Slavik L., Ulehlova J. (2021). Current promising biomarkers and methods in the diagnostics of antiphospholipid syndrome: a review. Biomedicines.

[22] Jiang D., Lim W., Crowther M., Garcia D. (2021). A systematic review of the association between anti-β-2 glycoprotein I antibodies and APS manifestations. Blood Adv.

[23] Meroni P.L., Borghi M.O., Raschi E., Tedesco F. (2011). Pathogenesis of antiphospholipid syndrome: understanding the antibodies. Nat. Rev. Rheumatol..

[24] Pal’keeva M.E., Sidorova M.V., Kuznetsova T.V., Kobylyanskii A.G., Tishchenko V.A., Nasonov E.L. (1996). Synthesis and antigenic properties of peptide fragments of β_2_ -glycoprotein-I. Russ. J. Bioorg. Chem.

[25] McDonnell T., Wincup C., Buchholz I. (2020). The role of beta-2-glycoprotein I in health and disease associating structure with function: more than just APS. Blood Rev.

[26] Hochberg M.C. (1997). Updating the American College of Rheumatology revised criteria for the classification of systemic lupus erythematosus. Arthritis Rheum.

[27] Korneenkov, A.A., Ryazantsev, S.V., and Vyazems-kaya, E.E., Calculation and interpretation of indicators of informativeness of diagnostic medical technologies, *Med. Council*, 2019, no. 20, pp. 45–51.

[28] Fletcher R., Fletcher S., Wagner E. (1998). *Clinical Epidemiology. Fundamentals of Evidence-Based Medicine*.

[29] Weaver, J.C. and Krilis, S.A., Giannakopoulos B. Oxidative post-translational modification of beta 2-glycoprotein I in the pathophysiology of anti-phospholipid syndrome, *Free Radical Biol. Med.*, 2018. 10.1016/j.freeradbiomed.2018.03.048 10.1016/j.freeradbiomed.2018.03.048 29604397

[30] de Groot P.G., Meijers J.C. (2011). β(2)-Glycoprotein I: evolution, structure and function. J. Thromb. Haemost..

[31] de Laat B., Derksen R.H., van Lummel M. (2006). Pathogenic anti-beta2-glycoprotein I antibodies recognize domain I of beta2-glycoprotein I only after a conformational change. Blood.

[32] Iverson G.M., Victoria E.J., Marquis D.M. (1998). Anti-beta2 glycoprotein I (beta2GPI) autoantibodies recognize an epitope on the first domain of beta2GPI. Proc. Natl. Acad. Sci. U. S. A..

[33] Mahler M., Norman G.L., Meroni P.L., Khamashta M. (2012). Autoantibodies to domain 1 of beta 2 glycoprotein 1: a promising candidate biomarker for risk management in antiphospholipid syndrome. Autoimmun. Rev..

[34] Chighizola C.B., Gerosa M., Meroni P.L. (2014). New tests to detect antiphospholipid antibodies: anti-domain I beta-2-glycoprotein-I antibodies. Curr. Rheumatol. Rep..

[35] Ioannou Y, Romay-Penabad Z, Pericleous C (2009). In vivo inhibition of antiphospholipid antibody-induced pathogenicity utilizing the antigenic target peptide domain I of beta2-glycoprotein I: proof of concept. J. Thromb. Haemost..

[36] Pericleous C., Ruiz-Limón P., Romay-Penabad Z., Marín A.C. (2015). Proof-of-concept study demonstrating the pathogenicity of affinity-purified IgG antibodies directed to domain I of β_2_-glycoprotein I in a mouse model of anti-phospholipid antibody-induced thrombosis. Rheumatology.

[37] de Laat B., Derksen R.H., Urbanus R.T., de Groot P.G. (2005). IgG antibodies that recognize epitope Gly40–Arg43 in domain I of beta 2-glycoprotein I cause LAC, and their presence correlates strongly with thrombosis. Blood.

[38] Colasanti T., Alessandri C., Capozzi A. (2012). Autoantibodies specific to a peptide of β_2_-glycoprotein I cross-react with TLR4, inducing a proinflammatory phenotype in endothelial cells and monocytes. Blood.

[39] Mondejar R., González-Rodríguez C., Toyos-Sáenz de Miera F.J. (2014). Role of antiphospholipid score and anti-β_2_-glycoprotein I Domain I autoantibodies in the diagnosis of antiphospholipid syndrome. Clin. Chim. Acta.

[40] Zhang S., Wu Z., Li P. (2015). Evaluation of the clinical performance of a novel chemiluminescent immunoassay for detection of anticardiolipin and anti-beta2-glycoprotein 1 antibodies in the diagnosis of antiphospholipid syndrome. Medicine.

[41] Zhang S., Wu Z., Chen S. (2016). Evaluation of the diagnostic potential of antibodies to beta2-glycoprotein 1 domain 1 in Chinese patients with antiphospholipid syndrome. Sci. Rep..

[42] Rodríguez-García V., Ioannou Y., Fernández-Nebro A. (2015). Examining the prevalence of non-criteria anti-phospholipid antibodies in patients with anti-phospholipid syndrome: a systematic review. Rheumatology.

[43] Tonello M., Mattia E., Del Ross T. (2018). Clinical value of anti-domain I-β_2_  Glycoprotein 1 antibodies in antiphospholipid antibody carriers. A single centre, prospective observational follow-up study. Clin. Chim. Acta.

[44] Liu T., Gu J., Wan L. (2020). Anti-β_2_GPI domain 1 antibodies stratify high risk of thrombosis and late pregnancy morbidity in a large cohort of Chinese patients with antiphospholipid syndrome. Thromb. Res..

[45] Liu T., Gu J., Wan L. (2020). “Non-criteria” antiphospholipid antibodies add value to antiphospholipid syndrome diagnoses in a large Chinese cohort. Arthritis Res. Ther..

[46] Pengo V. (2020). Additional laboratory tests to improve on the diagnosis of antiphospholipid syndrome. J. Thromb. Haemost..

