# Erratum to: **Antibodies to Domain I β2-Glycoprotein 1 in Patients with Antiphospholipid Syndrome and Systemic Lupus Erythematosus**

**DOI:** 10.1134/S160767292305006X

**Published:** 2024-01-24

**Authors:** T. M. Reshetnyak, F. A. Cheldieva, M. V. Cherkasova, S. I. Glukhova, A. M. Lila, E. L. Nasonov

**Affiliations:** 1 Nasonova Research Institute of Rheumatology, Moscow, Russia; 2 Russian Medical Academy of Continuous Professional Education of the Ministry of Healthcare of the Russian Federation, Moscow, Russia; 3 Sechenov First Moscow State Medical University of the Ministry of Health Care of Russian Federation (Sechenov University), Moscow, Russia

The article “Antibodies to Domain I β2-Glycoprotein 1 in Patients with Antiphospholipid Syndrome and Systemic Lupus Erythematosus”, written by T.M. Reshetnyak, F.A. Cheldieva, M.V. Cherkasova, S.I. Glukhova, A.M. Lila , and E.L. Nasonov, was originally published electronically in Springer-Link on October 13, 2023 without Open Access. After publication in volume 511, pages 219–226, the authors decided to make the article an Open Access publication. Therefore, the copyright of the article has been changed to © The Author(s) 2023 and the article is forthwith distributed under the terms of a Creative Commons Attribution 4.0 International License (http://creativecommons.org/licenses/by/4.0/, CC BY), which permits use, duplication, adaptation, distribution and reproduction of a work in any medium or format, as long as you cite the original author(s) and publication source, provide a link to the Creative Commons license, and indicate if changes were made.

